# Targeting glutamine metabolic reprogramming of SLC7A5 enhances the efficacy of anti-PD-1 in triple-negative breast cancer

**DOI:** 10.3389/fimmu.2023.1251643

**Published:** 2023-09-04

**Authors:** Renhong Huang, Han Wang, Jin Hong, Jiayi Wu, Ou Huang, Jianrong He, Weiguo Chen, Yafen Li, Xiaosong Chen, Kunwei Shen, Zheng Wang

**Affiliations:** Department of General Surgery, Comprehensive Breast Health Center, Ruijin Hospital, Shanghai Jiao Tong University School of Medicine, Shanghai, China

**Keywords:** triple-negative breast cancer, glutamine metabolism, SLC7A5, immunotherapy, synergic effect

## Abstract

**Background:**

Triple-negative breast cancer (TNBC) is a heterogeneous disease that is characterized by metabolic disruption. Metabolic reprogramming and tumor cell immune escape play indispensable roles in the tumorigenesis that leads to TNBC.

**Methods:**

In this study, we constructed and validated two prognostic glutamine metabolic gene models, Clusters A and B, to better discriminate between groups of TNBC patients based on risk. Compared with the risk Cluster A patients, the Cluster B patients tended to exhibit better survival outcomes and higher immune cell infiltration. In addition, we established a scoring system, the glutamine metabolism score (GMS), to assess the pattern of glutamine metabolic modification.

**Results:**

We found that solute carrier family 7 member 5 (SLC7A5), an amino acid transporter, was the most important gene and plays a vital role in glutamine metabolism reprogramming in TNBC cells. Knocking down SLC7A5 significantly inhibited human and mouse TNBC cell proliferation, migration, and invasion. In addition, downregulation of SLC7A5 increased CD8^+^ T-cell infiltration. The combination of a SLC7A5 blockade mediated via JPH203 treatment and an anti-programmed cell death 1 (PD-1) antibody synergistically increased the immune cell infiltration rate and inhibited tumor progression.

**Conclusions:**

Hence, our results highlight the molecular mechanisms underlying SLC7A5 effects and lead to a better understanding of the potential benefit of targeting glutamine metabolism in combination with immunotherapy as a new therapy for TNBC.

## Introduction

Triple-negative breast cancer (TNBC) is a subtype of breast cancer characterized by the negative expression of estrogen receptor (ER), progesterone receptor (PR) and human epidermal growth factor receptor-2 (HER-2), and it accounts for 15-20% of all breast cancers (1). Due to its aggressive nature, higher recurrence and mortality rates, and lack of therapeutic targets, TNBC is generally considered to be the subtype of breast cancer with the worst prognosis (2, 3). The clinical management of TNBC remains a great challenge because it is a highly heterogeneous disease that lacks specific therapeutic targets. To manage the disease, a stratification of TNBC risk and identification of new therapeutic targets for the precise treatment of TNBC are necessary.

Metabolic reprogramming is a hallmark of cancers (4). Cancer cells are in a hypermetabolic state, with changing metabolic pathways that meet their uncontrolled growth and proliferation needs. As research into cancer has advanced, increasing evidence suggested that altered metabolism, especially amino acid metabolism, in tumors provides potential targets for breast cancer treatment. Glutamine, the most abundant amino acid in human plasma, is an important nutrient for cancer cell growth and proliferation, providing essential nitrogen for nucleotide synthesis (5). The high demand for glutamine contributes to the glutamine-dependent phenotype in many aggressive cancers, including TNBC. The glutamine catabolic pathway is in a highly active state, which is important for amino acid-related metabolic shifts. Compared with other subtypes of breast cancer, TNBC is characterized by the overexpression of glutaminase (GLS) (6), which results in an increased rate of glutamine metabolism. A metabolomic analysis revealed a low level of glutamine and a high level of glutamate in TNBC cells, indicating the enhanced catabolism of glutamine (7). As an important rate-limiting enzyme in the glutamine metabolic pathway, GLS is expected to be a novel cancer treatment target (8). SLC7A5, also known as LAT1, to act as a chaperone and allow their localization to the plasma membrane (9). SLC7A5 is an amino acid transporter, and its expression in tumor cells contributes to the increased metabolism needed to meet cancer cell requirements. SLC7A5 is a critical transporter that mediates uptake of several essential amino acids in highly proliferative tumors and activated T cells (10). Moreover, previous studies have shown that SLC7A5 is important for the growth and proliferation of tumor cells (11). Studying the biological role of SLC7A5 in breast cancer may lead to its identification as a novel tumor biomarker and target in corresponding therapeutics (12).

In addition to metabolic reprogramming, immune escape of tumor cells plays an equally important role in tumorigenesis. In TNBC, the immune cell infiltration rate in the tumor microenvironment (TME) is high; therefore, an in-depth study into the altered immune microenvironment may lead to immunotherapy that better benefit TNBC patients. Related studies have shown that glutamine metabolic reprogramming exerts an impact on the TME and immune response. The predatory uptake of glutamine by tumor cells limits the pool of glutamine available for uptake by immune cells, inhibiting the antitumor immune response. A hypothesis of “glutamine steel” (8) has been proposed: It suggests that selectively blocking the metabolism of glutamine in tumor cells can attenuation the competition for glutamine and restore the immune response function of immune cells (13). Additionally, reprogramming of glutamine metabolism in tumor cells affects the immune response by regulating PD-L1 expression. *In vivo* experiments have shown that targeting glutamine metabolism in combination with treatment with a PD-L1 monoclonal antibody helped to enhance the antitumor immune response (14), making this combination a promising therapy.

## Methods

### Data acquisition and study design

In this study, the data from 2 appropriate breast cancer cohorts (GSE42568 and The Cancer Genome Atlas-Breast Invasive Carcinoma (TCGA-BRCA)) were collected for further analysis. The consolidated transcriptome expression matrix of breast cancer cells as well as clinical data were obtained from the Gene Expression Omnibus (GEO; http://www.ncbi.nlm.nih.gov/geo/) and The Cancer Genome Atlas (TCGA, https://www.cancer.gov/tcga) databases. Case data without survival information or survival status were excluded. A list of 17 genes encoding proteins participating in glutamine metabolic pathways reported in previous studies was compiled (15). We identified these genes as glutamine metabolic genes (GMGs). The GSE37751, GSE13908, GSE13908, GSE21653 and GSE25066 datasets were utilized to validate the clinicopathological characteristics of SLC7A5 in breast cancer samples.

### Identification of the prognostic characteristics of glutamine metabolic genes

Differentially expressed GMGs between breast cancer samples and normal samples were identified and confirmed with the Wilcoxon test and R package limma. For each differentially expressed gene, the highest cutoff for the mRNA expression level obtained with the “surv_cutpoint” command was used to classify patients into a high-expression-level group and a low-expression-level group. The outcome differences were calculated with the log-rank test via the Kaplan−Meier (KM) method. The R packages KMsurv, survival, and survminer were utilized to perform a prognostic analysis. A P value<0.05 was considered to be statistically significant.

### Establishment and functional enrichment analysis of glutamine metabolic clusters

Considering the expression of the 17 GMGs, we utilized the ConsensuClusterPlus (16) package to identify disparate GMG modification patterns and classified patients for further analysis. The datasets were clustered via the Euclidean squared distance metric and a K-means algorithm with a k ranging from 2 to 9. The results were presented in the form of heatmaps on the basis of the consistency matrix generated by the R package pheatmap. The KM curve shows the overall survival (OS) for each glutamine metabolic cluster. In addition, the log-rank test was used to evaluate the survival difference among the clusters with a criterion of a P value< 0.05. To further explore the biological characteristics of clusters, we utilized gene set variation analysis (GSVA) to evaluate biological pathways between among clusters with the R package GSVA (17) and “c2.cp.kegg.v7.4.symbols” from the Molecular Signatures Database (MSigDB). According to the criteria of an |logFC|≥2 and adj.P.Val ≤ 0.01, the different genes among clusters were screened. Then, the enrichment of Gene Ontology (GO) terms and Kyoto Encyclopedia of Genes and Genomes (KEGG) pathways was determined to perform a functional enrichment analysis.

### Identification of the immune cell characteristics in glutamine metabolic clusters

Gene expression profile data were used to determine the relative degree of immune cell infiltration in individual tumor samples via ssGSEA. In addition, the stromal cell scores and immune cell scores in the merged dataset (TCGA-BRCA dataset and GSE42568) were calculated with the ESTIMATE algorithm to determine the ESTIMATE score for each breast cancer sample. A Wilcoxon test was then performed to determine the significance of differences in immune cell abundance and checkpoint levels among different clusters. A P value< 0.05 was considered to be statistically significant.

### Establishment of the glutamine metabolism score

To quantify the pattern of glutamine metabolic modification in individual tumors, we constructed a scoring system to assess the pattern of glutamine metabolism modification—the glutamine metabolism score (GMS)—in individual breast cancer patients. The steps for establishing the GMS were as follows: First, we performed a prognostic analysis of 17 GMGs via univariate Cox regression. According to the P<0.05 level of significance, genes that were significantly related to prognosis were identified and further analyzed. The GMS was then determined via principal component analysis (PCA). Principal Component 1 and principal Component 2 were selected as signature scores. The advantage of this approach was that the score of the group with the largest set of closely associated (or anticorrelated) genes was the focus, in contrast to the smaller contributions from genes that were not included in a group with closely related genes. We then defined the GMS utilizing a method similar to that used for establishing a GGI (18, 19):


$$\text{GMS}=\sum \left(\text{PC}1_{i}+\text{PC}2_{i}\right)$$

where i represents the expression of a GMG.

### Identification of the clinicopathological characteristics of the glutamine metabolism score

Considering the median GMS expression, we classified all breast cancer samples into high- and low-GMS groups. The KM curve was used to evaluate the prognostic features of the GMS with the survminer package. Then, the ggplot2 package and a Wilcoxon test were used to analyze the differential expression of GMSs among diverse clinicopathological characteristics, including stage and TNM stage. In addition, we employed the ggplot2 package to plot the proportions of various clinicopathological characteristics in the high and low GMS groups and analyzed differences by chi-square test. In addition, we explored the clinical prognostic features related to the GMS. On the basis of the GMS at different clinicopathological stages, the “surv_cutpoint” command was used to select the best threshold discrimination value, and the patients were thus assigned to the high GMS group and the low GMS group. The KM curve presents the differences in survival between the high and low GMS groups at different clinicopathological stages.

### Correlation analysis of SLC7A5 in breast cancer patients

The breast cancer patients for which clinical data were available were assigned to the low- and high-risk groups according to the median SCL7A5 level. The ggplot2 package and chisq test were used to plot the percentages of patients in the high and low SCL7A5 groups at different clinicopathological stages. In addition, to further investigate the correlation between SLC7A5 expression level and clinicopathological characteristics, we validated the differential expression of SLC7A5 at various clinicopathological stages in multiple TCGA and GEO datasets. In addition, we investigated differences in drug sensitivity between the high and low SLC7A5 groups. A Wilcoxon test was performed to analyze the differences in the half maximal inhibitory concentration (IC50) of drugs between the high and low SLC7A5 groups. We also explored the correlation between SLC7A5 levels and immune factors, including immune cell infiltration and common immune checkpoints. A gene set enrichment analysis (GSEA) was performed to analyze differences in the activated biological pathways between the high and low SLC7A5 groups.

### Cell culture

The MDA-MB-231 human and 4T1 mouse TNBC cell lines and were all obtained from the Type Culture Collection of the Chinese Academy of Sciences (Shanghai, China). The MDA-MB-231 cells were cultured in high-glucose DMEM (Sigma−Aldrich, USA) supplemented with 10% fetal bovine serum (FBS) (Gibco, Thermo Fisher Scientific, Inc.) and 1% antibiotic (mixtures of penicillin and streptomycin, Beyotime, Shanghai), while the 4T1 cells were cultured in Roswell Park Memorial Institute (RPMI) 1640 (Gibco, Thermo Fisher Scientific, Inc.) containing 10% FBS and 1% antibiotic with an atmosphere of 37°C and 5% CO_2_.

### Cell transfection

Cell transfection was performed according to the guidance of a technician. Briefly, the RNA interference sequence, a short hairpin RNA (shRNA) targeting SLC7A5, was cloned into a linearized pLKO.1 plasmid (BioGene Co., Ltd., Shanghai) to knock down SLC7A5 expression. The recombination lentiviral plasmid, along with the packaging plasmids psPAX2 and pMD2G, was transfected into 293T cells. After transfection for two days, recombined lentiviral virus was collected and used to transduce cells in specific groups. Cells transduced with pLKO.1-scramble shRNA were the negative control cells.

### Cell viability assay

Cell viability was measured with a CellTiter-Glo^®^ Luminescent Cell Viability Assay (Promega, Cat # G7572, Lot # 268755) in accordance with the manufacturer’s instructions. Briefly, cells were seeded onto 96-well plates at a density of 4×10^3^/well, followed by incubation with a 66-µl solution for one hour. Cell viability was quantified based on the OD450, which was obtained with a microplate reader.

### Wound healing, cell migration and invasion assay

A scratch/wound healing assay was performed to measure the cell migration rate. Cell migration and invasion abilities were determined via Transwell assay. The experimental procedures were described in our previous study (20). For wound healing assay, a total number of 5×105 cells were seeded onto 6-well culture plates, and we used a sterile micropipette tip to scratch a confluent monolayer. The wounds of the cells were allowed to cover, and graphs of the same wound were recorded. The wound rates were calculated by ImageJ software (version 1.5.3). For transwell assays, cell migration and invasion were measured with Transwell assay (24-well insert, 8 μm pore size; BD Biosciences, Bedford, MA, USA). The filters (Corning Inc., USA) were coated with or without 55 μL of Matrigel for invasion and migration, respectively. Then, 104 cells were suspended in in the upper chamber with serum-free medium. Then, 500 μl of 10% FBS supplemented media were added to the bottom chamber. Cells were fixed with 4% paraformaldehyde for 30 min and stained with 0.1% crystal violet for 30 minutes. Finally, number of invasion and migration cells were counted.

### Western blotting

Proteins were separated by sodium dodecyl sulfate−polyacrylamide gel electrophoresis (SDS−PAGE) and transferred onto polyvinylidene difluoride membranes (PVDF). Subsequently, the membranes were blocked for approximately 1 h with 5% nonfat dry milk dissolved in TBST buffer and washed three times in TBST for 10 min each time. In addition, the membranes were incubated with primary antibodies at 4°C overnight (anti-SLC7A5, 1:1000, CST; anti-E-cadherin, 1:1000, CST; anti-N-cadherin, 1:1000, CST; anti-vimentin, 1:1000, CST; anti-CD8^+^T, 1:1000, CST; and anti-snail, 1:1000). The membranes were incubated with secondary antibodies at room temperature for 1 h. GAPDH was used as the loading control and for expression level normalization (anti-GAPDH, 1:1000, Abcam). The membranes were imaged using a Luminescent Image Analyzer detection system (Fujifilm, LAS-4000).

### Immunofluorescence

The cells were seeded on a coverslip at a density of 1×10^6^ per well. Cells on coverslips were fixed with 4% paraformaldehyde for 20 min, permeabilized with 0.2% Triton X-100 for 20 min, blocked with 5% BSA for 60 min and stained with primary antibody overnight at 4°C. Then, an Alexa 488-coupled goat anti-rabbit IgG secondary antibody was incubated with the cells for 1 h in the dark. DAPI was used to label the nuclei for 20 min. Finally, we photographed the cells using a fluorescence microscope.

### *In vivo* tumor model and combined therapy model

Four- to five-week-old female BALB/c mice were obtained from the Shanghai Laboratory Animal Company. All the experimental procedures in this study were performed according to the animal ethics guidelines approved by the Medical Ethics Committee of Ruijin Hospital, Shanghai Jiao Tong University. A total of 1×10^6^ 4T1 cells transduced with an shNC or sh-SLC7A5 vector were subcutaneously injected into the fat pad of the mice (n=6 each group). We measured and recorded the tumor size and weight of the mice every four days. For the combined therapy model, anti-PD-1 antibody (10 μg once) and JPH203 (10 μg once) were injected every two days after the tumor volume reached 50 mm^3^. In the fourth week, the mice were sacrificed, and the tumor xenografts and lungs were resected for tumor IHC and HE analysis.

### Immunohistochemistry and tissue microarray

Paraffin-embedded tissue sections were cut into 4-μm-thick sections for use in an IHC analysis. The staining was performed overnight via incubation with primary antibodies (anti-SLC7A5, 1:200, CST; anti-E-cadherin, 1:200, CST; anti-N-cadherin, 1:200, CST; anti-Vimentin, 1:200, CST; anti-CD8^+^T, 1:200, CST; and anti-CD4^+^T, 1:200, CST), followed by incubation with secondary antibody for an hour at room temperature. The immunohistochemical analysis was performed and the results were evaluated by two individual pathologists without knowledge of the clinical or pathological characteristics of the patients. Immunoreactivity was evaluated using the H-score system by at least two investigators according to the percentage of positively stained cells and the intensity of staining. Details for IHC were the same as our study previously described (21). The percentage score of positive staining cells was defined: 0,< 1%; 1, 1–25%; 2, 26–50%; 3, 51–75%; and 4, > 75%. Furthermore, the staining intensity score was defined as follows: 0, negative; 1, weak positive; 2, moderate positive; and 3, strong positive. Based on the immunoreactivity scores, patients in the tissue microarray were categorized into the low-expression and high-expression groups, which were then analyzed to determine the overall survival and progression-free survival.

### Hematoxylin and eosin staining

The lungs derived from sacrificed mice were stained with an HE staining kit (Beyotime Biotechnology, Shanghai, China). Briefly, tissues were fixed with 4% paraformaldehyde and dehydrated in an ethanol gradient. The tissues were cleared with xylene, embedded in paraffin, cut into 5-μm sections, dewaxed, dehydrated, and then stained with hematoxylin for 5 min and with eosin for 3 min. A microscope was used to evaluate the morphology of the metastatic nodules in the lung.

### Statistical analysis

All analyses were performed with R 4.1.0. All statistical tests were two-sided, and a P value<0.05 was considered to be statistically significant unless otherwise noted. The relationship between hub genes and overall survival was analyzed with the KM curve, which had been established on the basis of log-rank tests. The univariate regression model was used to analyze the effects of individual variables on survival. The asterisks represent the statistical p value (*p<0.05, **p<0.01, ***p<0.001).

## Results

### Establishment and biological characteristics of glutamine metabolism clusters

**Figure 1:**
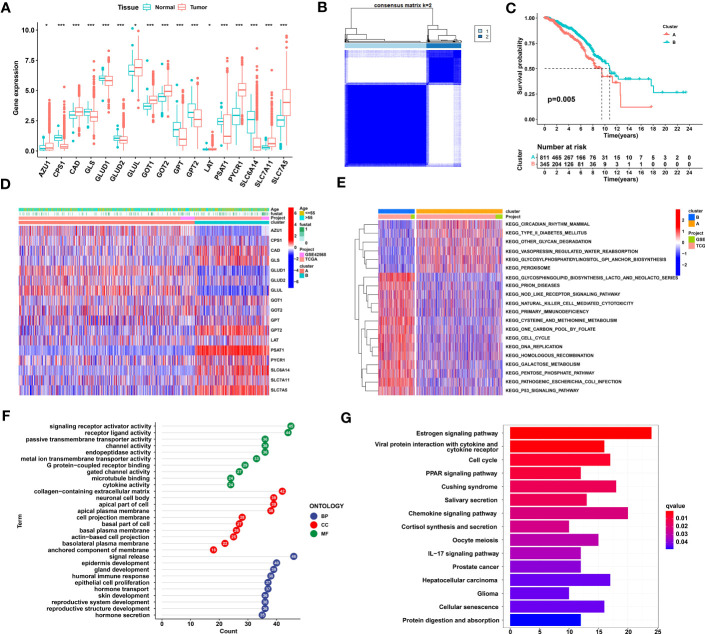
Identification and comparison of glutamine metabolic clusters in TNBC. **(A)** Differential expression of 17 glutamine metabolism genes between normal and breast cancer tissues. **(B)** Consensus clustering analysis led to the identification of the consensus clustering matrices for k = 2. **(C)** Kaplan−Meier curve showing that the overall survival of the patients in Cluster B was significantly longer than that of the patients in Cluster **(A, D)** Heatmap showing gene expression and clinical correlation between Clusters A and B **(E)** A GSVA showing the activation status of biological pathways on the basis of different glutamine metabolism patterns. Heatmaps were used to visualize these biological processes, with red representing activated pathways and blue representing inhibited pathways. **(F)** GO enrichment analysis of differentially expressed glutamine metabolic genes. **(G)** KEGG enrichment analysis of differentially expressed glutamine metabolic genes. TNBC, Triple-negative breast cancer; GSVA, gene set variation analysis; GO, Gene Ontology; KEGG, Kyoto Encyclopedia of Genes and Genomes. The asterisks represent the statistical significance as determined by the *P* value (*P< 0.05, ***P< 0.001).

In total, 17 GMGs were evaluated in our study, and the expression levels of all GMGs varied between tumor and normal tissues. Among these GMGs, 9 genes were highly expressed in tumor tissue, and 8 were expressed at low levels in tumor tissue (**Figure 1A**). In addition, we explored the relationships between GMGs and the prognosis of breast cancer patients. As shown in **Figure S1**, the expression levels of 14 differentially expressed GMGs were mostly related to the prognosis of breast cancer patients. Then, based on the mRNA expression levels of these 17 GMGs in the merged dataset (TCGA-BRCA and GSE42568), the breast cancer samples were classified into 2 distinct clusters by utilizing Consensus Cluster Plus (**Figure 1B**). The KM curve reveals the survival difference between the two clusters, and Cluster B showed a greater survival advantage (**Figure 1C**). **Figure 1D** shows the distribution of gene expression levels and clinicopathological characteristics in different clusters. To identify the underlying mechanism leading to different prognoses between Clusters A and B, we explored biological pathway differences between the two glutamine metabolism patterns that distinguish these groups. GSVA revealed that a variety of cancer-promoting and metabolic pathways were enriched in Cluster B; these pathways included the P53 signaling pathway, cell cycle, nod-like receptor signaling pathway, and cysteine and methionine metabolism (**Figure 1E**). Then, according to the criteria of |logFC|≥2 and adj.P.Val ≤ 0.01, 120 differentially expressed genes between Clusters A and B were identified. The results of a GO analysis indicated that in the biological process (BP) category, genes were mainly enriched in the process of cell development and the process of hormone secretion and transport. In the cellular components (CC) category, genes were mainly concentrated in the apical part and basal part of the cell. In addition, in the molecular functions (MF) category, genes were significantly focused on transmembrane transporter activity and receptor−ligand activity (**Figures 1E, F**). The results from a KEGG analysis indicated that genes were mainly concentrated in PPAR signaling pathway, chemokine signaling pathway, and cell cycle (**Figure 1G**).

### TME and immune cell characteristics of glutamine metabolism clusters in patients with TNBC

**Figure 2:**
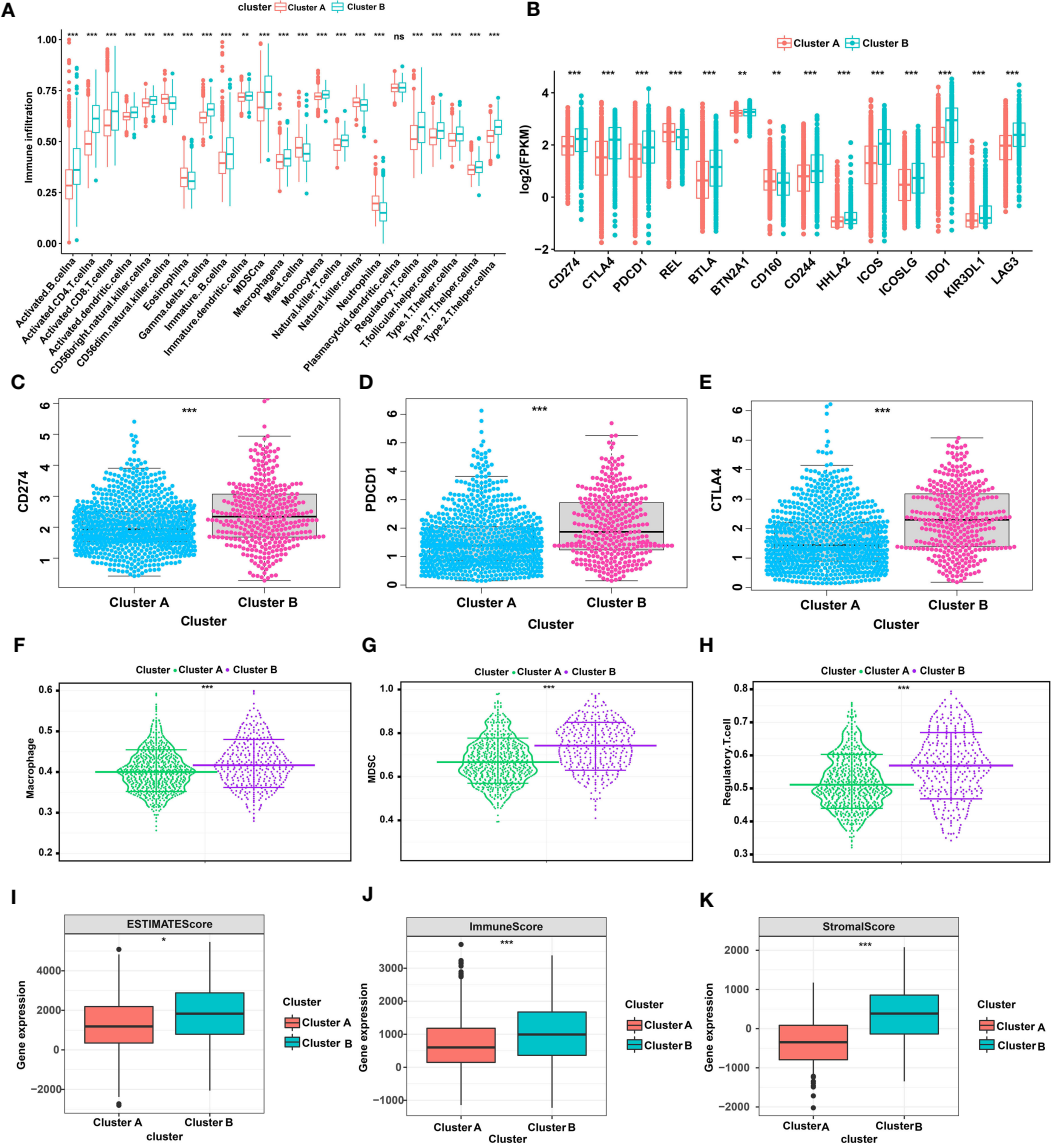
TME and immune cell infiltration characteristics in different glutamine metabolic clusters of TNBC. **(A)** Differential expression of 23 immune cells between Cluster A and Cluster B **(B)** Differential expression of 14 immune checkpoints between Clusters A and B **(C-E)** Differential expression of CD274, PDCD1 and CTLA4 between Cluster A and Cluster B **(F-H)** Differential abundance of different types of immunosuppressive cells between Cluster A and Cluster B (macrophages, MDSCs, and regulatory T cells). **(I-K)** The expression difference in the tumor microenvironment score between Cluster A and Cluster B (estimate score, immune score, and stromal score). The asterisks represent a statistically difference as indicated by *P* value (*P< 0.05, **P< 0.01, ***P< 0.001). TME, tumor microenvironment; TNBC, triple-negative breast cancer; CD274, cluster of differentiation 274; PDCD1, programmed cell death protein 1; CTLA4, cytotoxic T-lymphocyte associated protein 4; MDSC, myeloid-derived suppressor cells. NS, Not statistically significant.

Next, we further analyzed differences in immune cells, immune checkpoints, and the tumor microenvironment (TME) between glutamine metabolism clusters. First, we performed an ssGSEA to evaluate the abundance of immune cells in each single breast cancer sample and analyzed the differences in the types of immune cells between clusters. The results revealed that 23 kinds of immune cells were generally different between Cluster A and Cluster B, with immune cells more abundant in Cluster B (**Figure 2A**). In addition, as shown in **Figure 2B**, the levels of 14 immune checkpoints were significantly different between Clusters A and B. Specifically, the expression levels of PDCD1, CTLA4 and CD274 in Cluster B were all higher than those in Cluster A (**Figures 2C–E**). Moreover, MDSCs, regulatory T cells, and macrophages were highly abundant in Cluster B compared to Cluster A (**Figures 2F–H**). Regarding the tumor microenvironment scores, the ESTIMATE score, immune score and stromal score were higher in Cluster B than in Cluster A (**Figures 2I–K**). In addition, we analyzed the prognostic role played by 14 glutamine metabolic genes, as indicated when in the TCGA-BRCA and GSE42568 databases were merged, and the glutamine metabolism genes were found to be closely correlated with survival in patients with TNBC (**Figure S1**). Taken together, these results suggested that Cluster A was closely associated with immunosuppressive cells and may be involved in establishing an immunosuppressive TME, which may account for the poor prognosis of patients identified in Cluster A.

### Clinicopathological characteristics of the glutamine metabolism score in TNBC

**Figure 3:**
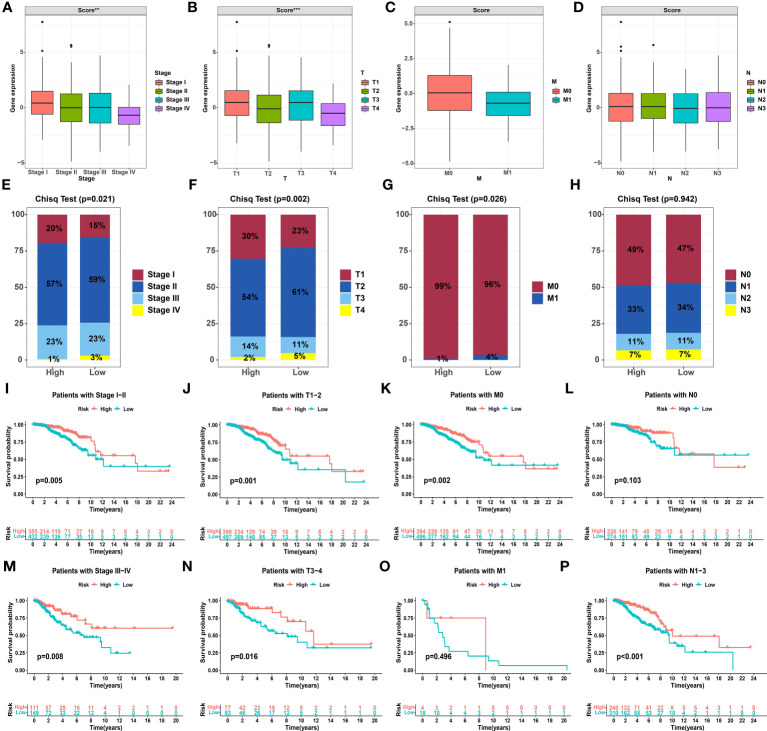
Correlation between the glutamine metabolism score and clinicopathological characteristics. **(A-D)** Differential glutamine metabolism scores at different clinicopathological stages. **(E-H)** Proportion plots presenting the distribution of different clinicopathological stages in the high and low glutamine metabolism score groups. **(I-P)** Kaplan−Meier analysis showing the overall survival based on the glutamine metabolism score at different clinicopathological stages. The asterisks represent the statistical *P* value (**P< 0.01, ***P< 0.001).

Due to the personal heterogeneity and complexity of GMG modifications, we constructed GMSs to assess GMG modification patterns in individual patients. Thus, a univariate analysis was performed to identify GMGs associated with prognosis. Ultimately, 5 genes (AZU1, GLUL, GOT2, PYCR1, and SLC7A5) were identified to establish the GMS (**Figure S2A**). Then, the KM curve revealed that the high-GMS group showed greater survival advantages than the low-GMS group (**Figure S2B**). We further analyzed the differential GMSs at different clinicopathological stages. We found that the GMS was significantly different in stage and T stage, and the later the stage was, the lower the GMS (**Figures 3A–D**). The percentage plot for the different clinicopathological stages indicates that the proportion of patients in advanced pathological stages in the low-GMS group was higher than the proportion of patients in the high-GMS group (**Figures 3E–H**). Next, a survival software package was used to validate the prognostic characteristics related to the GMS in different clinicopathological stages. The KM curve reveals that the high and low GMS groups showed significant differences by pathological stage and at the T stage, M0 stage and N1-3 stage, with the high GMS group exhibiting a higher survival rate (**Figures 3I–P**).

### Validation of the associations between clinical characteristics and SLC7A5 expression in TNBC

**Figure 4:**
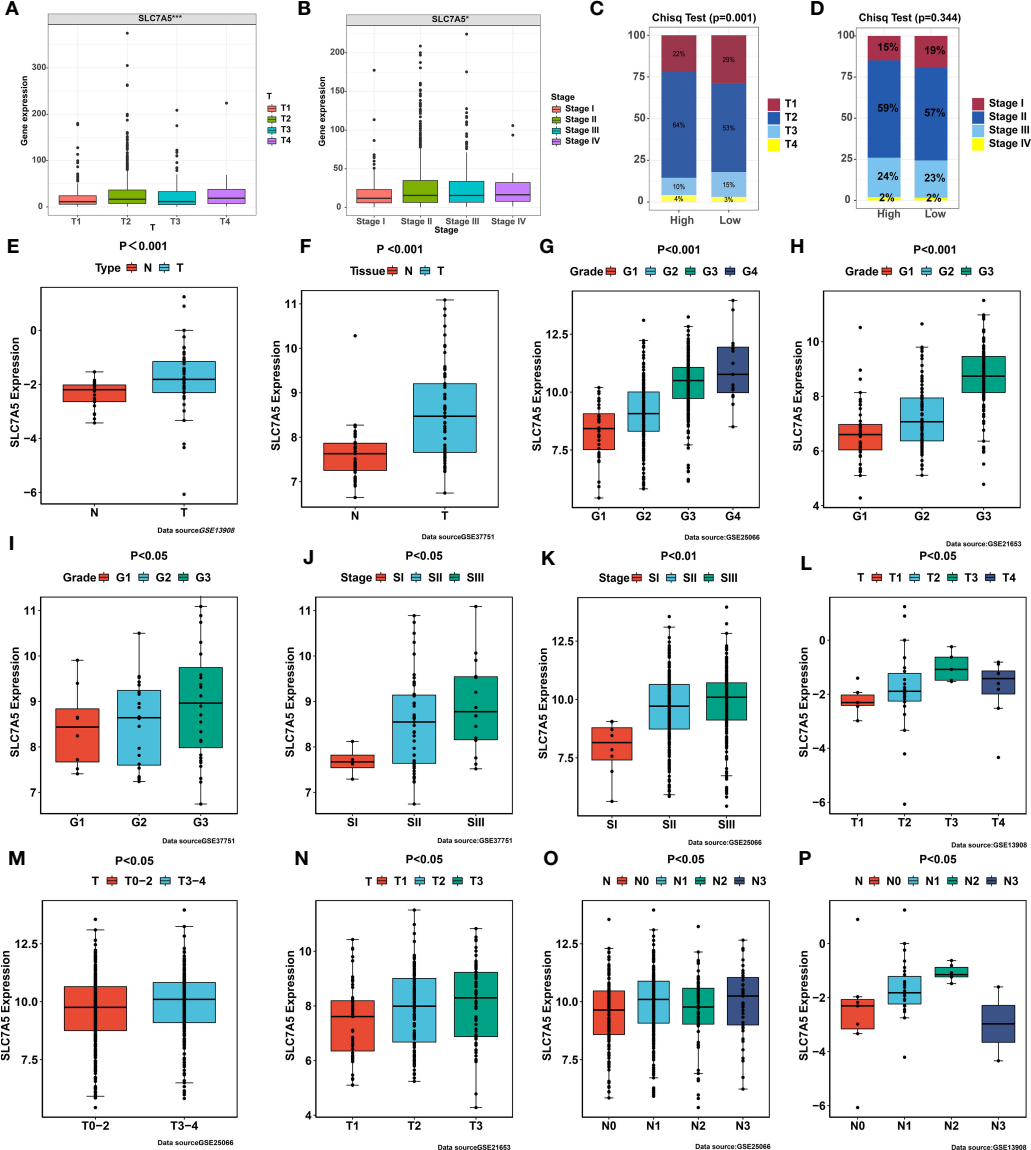
Correlation between SLC7A5 and clinicopathological features. **(A, B)** Expression levels of SLC7A5 at different clinicopathological stages. **(C, D)** Differences in the proportion of patients at different clinicopathological stages between high SLC7A5 and low SLC7A5expression subgroups **(E, F)** SLC7A5 expression was significantly higher in tumor tissues than in normal tissues in the GSE13908 and GSE37751 cohorts. **(G–I)** SLC7A5 expression was significantly higher in high-grade tumors than in low-grade tumors in the GSE25066, GSE21653, and GSE33751 cohorts. **(J, K)** SLC7A5 expression was significantly higher in the late TNM stages compared to early TNM stages in the GSE37751, GSE25066 and GSE66272 cohorts. **(L–P)** SLC7A5 expression was significantly higher in the late T and N stages than in the early T and N stages in the GSE21653, GSE25066 and GSE13908 cohorts. The asterisks represent the statistical significance as determined by the *P* value (*P< 0.05, ***P< 0.001).

**Figure 5:**
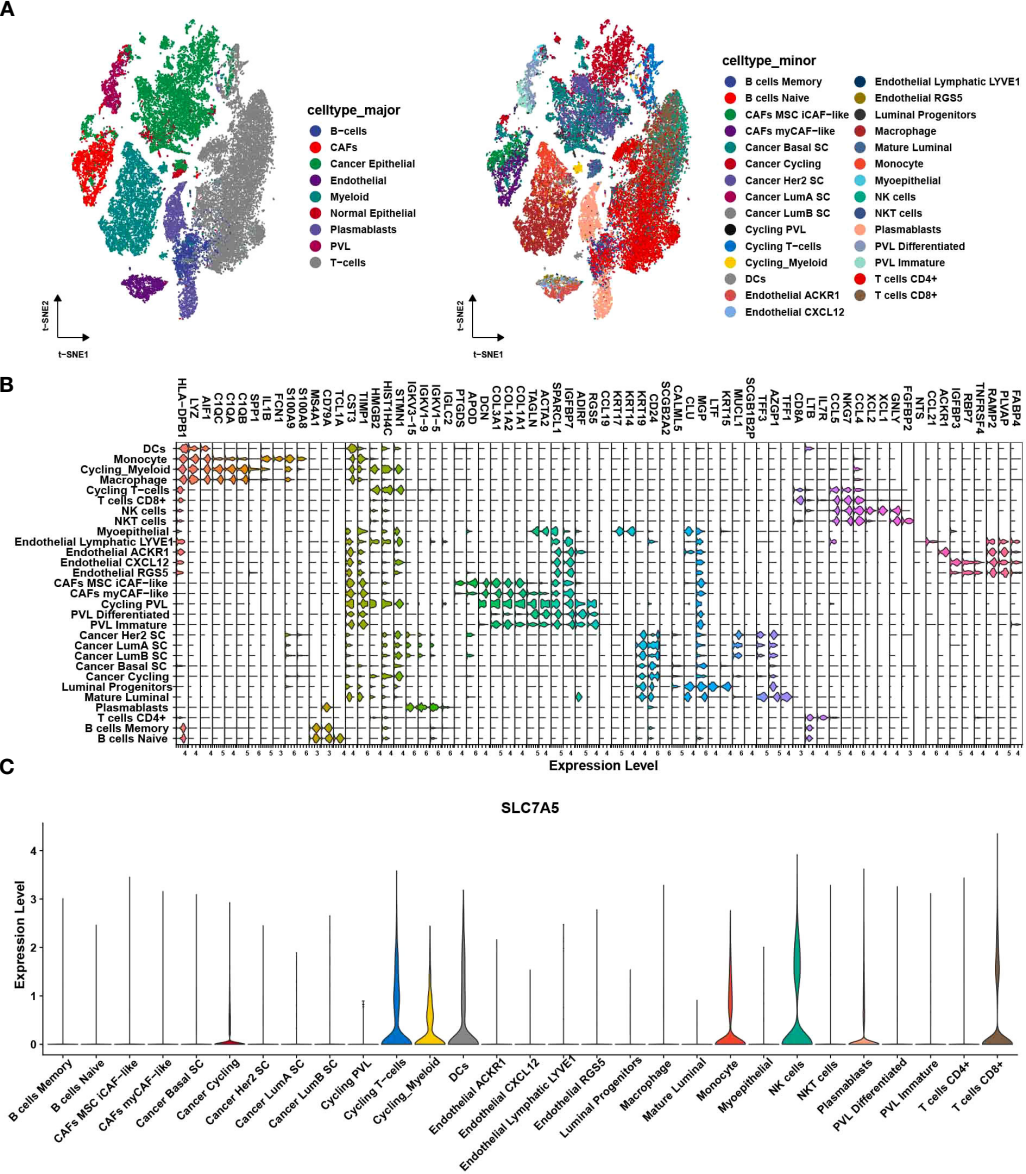
Single cell analysis of SLC7A5 in TNBC. **(A)** tSNE plot showing major (left) and minor cell type in TNBC tumor samples. **(B)** Violin plot showing top 3 expressed biomarkers in each minor cell cluster. **(C)** Violin plot showing expression level of SLC7A5 across different cell subclusters. TNBC, Triple-negative breast cancer.

To validate the role of SLC7A5 in breast cancer development, we analyzed the clinical relevance of SLC7A5 in multiple datasets. In the merged dataset (TCGA-BRCA and GSE42568), high SLC7A5 expression levels were significantly associated with the severity of the clinical predictors, namely, pathological stage (P< 0.001) and T stage (P< 0.05) (**Figures 4A, B**). We divided patients into high- and low-expression groups based on the median expression of SLC7A5 in the merged dataset (TCGA-BRCA and GSE42568) and analyzed the differences in the percentages of patients between the two groups on the basis of different pathological and T stages. The results indicated that the proportions of the patients at advanced pathological stage and T stage were higher in the high-SLC7A5 group (**Figures 4C, D**). We performed the same calculation with the GSE13908, GSE13908, GSE21653, GSE25066 and GSE37751 datasets. The outcomes of the analyses revealed that the expression of SLC7A5 was significantly increased in patients with breast cancer (**Figures 4E, F**), and the higher the expression of SLC7A5 was, the more advanced the tumor progression and the less differentiated the tumor cells (**Figures 4G–P**). To investigate the importance of SLC7A5 in TNBC tumor tissue, we performed single cell analysis based on scRNA-seq datasets of TNBC from public database (Combined Single-Cell and Spatial Transcriptomics Reveal the Metabolic Evolvement of Breast Cancer during Early Dissemination GSE176078). There were nine major cell types according to the classic cell reference information, and detailed subclusters of each cell type were also annotated (**Figures 5A**). Top 3 expressed biomarkers of each cell types were illustrated in **Figure 5B**. Furthermore, we analyzed the expression level of SLC7A5 in different subclusters. SLC7A5 was highly expressed in immune cells, including CD8+ T cells, cycling T cells, NK cells and dendritic cells (**Figure 5C**). To explore potential interactions between cancer cells with different expression level of SLC7A5 and other cells in TNBC, we divided cancel cells into SLC7A5-positive (count>0) and SLC7A5-negative cancer cells (count=0) based on the expression level of SLC7A5. Interestingly, the interaction weights between SCL7A5-positive cancer cells and other cells were significantly higher than that of SLC7A5-negative cancer cells (**Figure S3A**), indicating that SLC7A5 might led a potential role in cell communication. In addition, we noticed that some ligand-receptor pairs, including integrin, ERK, Notch signaling pathways, might involve in communication between SLC7A5-positive cancer cells and other immune cells (**Figure S3B**).

### The role of SLC7A5 in the drug sensitivity, immune characteristics, and survival of patients with TNBC

**Figure 6:**
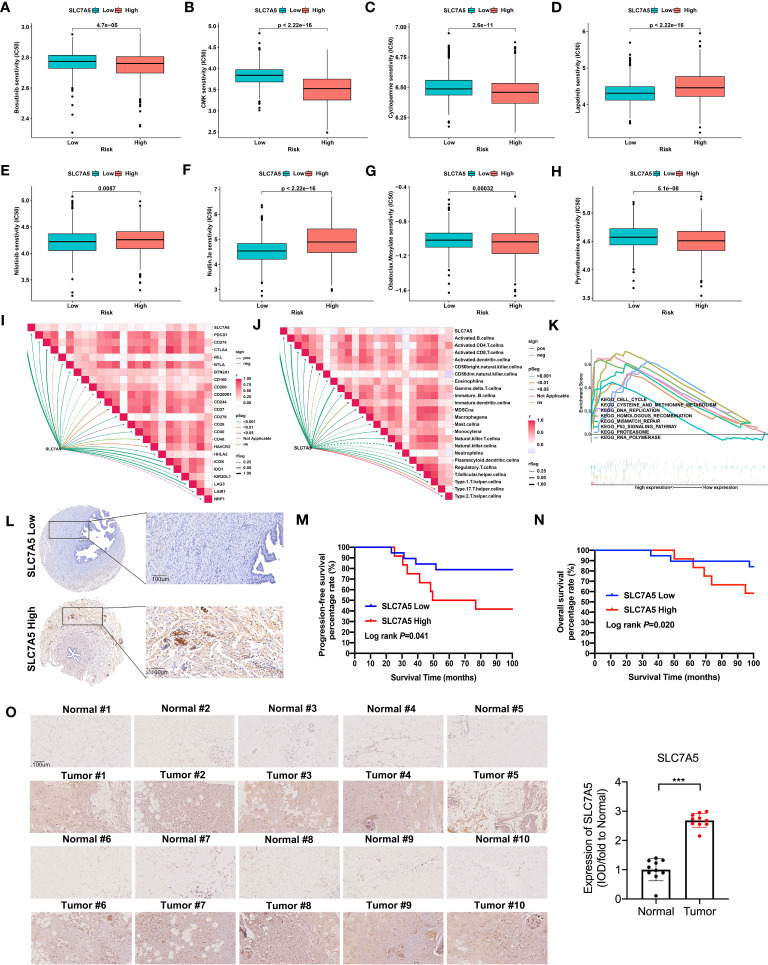
Drug sensitivity and immune cells characteristics based on SLC7A5 expression in patients with TNBC. **(A–H)** The IC50 values of 8 common drugs were significantly different between the high and low SLC7A5 expression groups. **(I–J)** Correlation of SLC7A5 expression with immune cell types and immune checkpoint expression. **(K)** GSEA enrichment analysis of SLC7A5 using TCGA-BRCA and GSE42568 datasets. P53 signaling pathway, cell cycle, and cysteine and methionine metabolism were differentially enriched between the high and low SLC7A5 groups. **(L)** Representative immunochemistry staining for SLC7A5 in a TNBC tissue microarray (Scale bar: 100 µm). **(M, N)** Kaplan–Meier survival curves showing PFS and OS according to high and low SLC7A5 expression levels. **(O)** Representative image showing immunochemistry staining of SLC7A5 and the and statistical analysis of SLC7A5 expression in 10 TNBC tumors and their adjacent tissues (scale bar: 100 µm). IC50, half-maximal inhibitory concentration; TCGA-BRCA, the Cancer Genome Atlas Breast Invasive Carcinoma; TNBC, triple-negative breast cancer; OS, overall survival; PFS, progression-free survival. The asterisks represent the statistical significance as determined by the *P* value (***P< 0.001).

To better guide clinical treatment strategies, we further compared the difference in response to drugs between the high- and low-SLC7A5 groups and found that the differences for eight drugs were statistically significant. Compared with the low-SLC7A5 group, the IC50 estimates for 5 drugs (bosutinib, CMK, bosutinib, pyrimethamine obatoclax and mesylate) were lower in the high-SLC7A5 group, while the IC50 estimates were higher for lapatinib, nilotinib and nutlin-3a (**Figures 6A–H**). These results suggested that the transcriptional level of SLC7A5 may affect the resistance and sensitivity of TNBC cells some common drugs. To further investigate the immune characteristics affected by SLC7A5, we analyzed the correlation between SLC7A5 level, immune cell type and immune checkpoint levels. The expression of SLC7A5 was significantly different and positively correlated with most immune cell types and checkpoint levels (**Figures 6I–J**). In addition, the results of a GSEA revealed that multiple cancer-promoting pathways and protein metabolism-related pathways were enriched in the high-SLC7A5 group, including the P53 signaling pathway, cell cycle, and cysteine and methionine metabolism (**Figure 6K**).

To obtain insight into how SLC7A5 expression levels might be linked to the survival of patients with TNBC, we evaluated the prognostic utility of SLC7A5 protein expression with a TNBC tissue microarray (**Figure 6L**). Notably, among TNBC patients, those with low-SLC7A5-expressing tumors showed a significantly better DFS (*log rank P*=0.041) and OS (*log rank P*=0.020) than those with high-SLC7A5-expressing tumors, which indicates that SLC7A5 plays an oncogene role and is a suitable prognostic factor for patients with TNBC (**Figures 6M, N**). Furthermore, we also detect the expression of SLC7A5 in 10 fresh triple-negative breast cancer issue and their comparing nontumor-adjacent tissue, and it was revealed that SLC7A5 was higher expression in tumor tissues than nontumor-adjacent tissues (**Figure 6O**).

### SLC7A5 knockdown attenuated tumor metastasis and inhibited T-cell infiltration in TNBC

**Figure 7:**
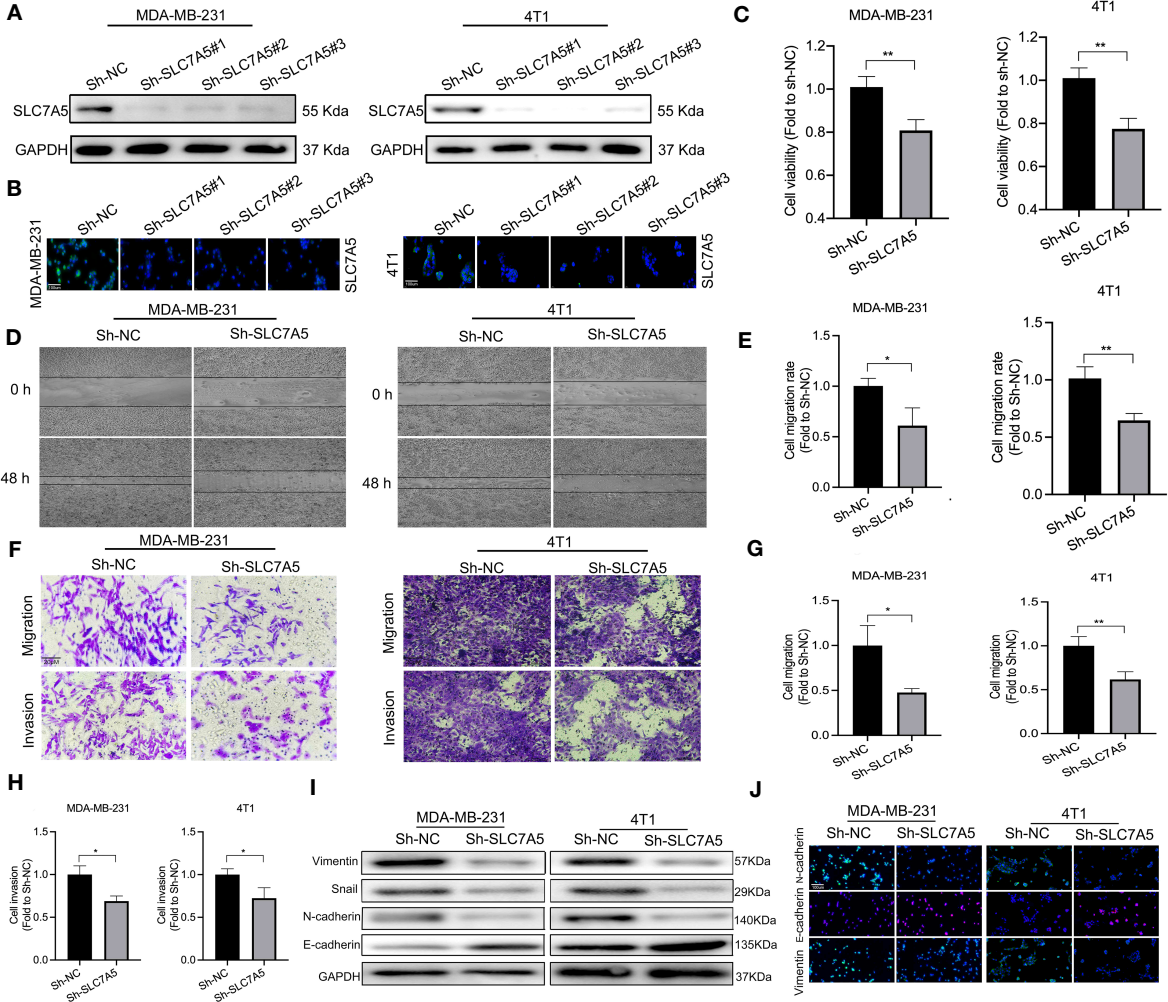
SLC7A5 promotes TNBC progression and the EMT *in vitro*. **(A, C)** Western blot and immunofluorescence analyses showing the expression of SLC7A5 in MDA-MB-231 and 4T1 cells (sh-NC, sh-SLC7A5#1, sh-SLC7A5#2 and sh-SLC7A5#3) (scale bar: 100 µm). **(B)** Viability of MDA-MB-231 and 4T1 cells after SLC7A5 knockdown. **(D, E)** Representative images showing wound healing of MDA-MB-231 and 4T1 cells after SLC7A5 gene knockdown and the statistical analysis of the results. **(F–H)** Representative images showing Transwell assays withMDA-MB-231 and 4T1 cells after SLC7A5 gene knockdown and the statistical analysis of the results(scale bar: 20 µm). **(I, J)** Western blot and immunofluorescence analysis of EMT marker expression after SLC7A5 knockdown (scale bar: 100 µm). TNBC, triple-negative breast cancer; EMT, epithelial-mesenchymal transition. The asterisks represent the statistical significance as determined by the *P* value (*P< 0.05, **P< 0.01).

To determine the manner and effect of SLC7A5 expression via a possible functional role and related biological activity in TNBC, we knocked down the SLC7A5 gene and suppressed its expression in the MDA-MB-231 and 4T1 cell lines. Western blotting and immunofluorescence assays were performed to determine the efficiency of SLC7A5 RNA knockdown via lentiviral transfection, and the results demonstrated that the SLC7A5 gene was successfully knocked down (**Figures 7A, B**). We then explored the possible role played by SLC7A5 in the proliferation, invasion, and metastasis of TNBC. In both the MDA-MB-231 and 4T1 TNBC cell lines, the results of the viability experiments showed that the cell proliferation ability was significantly suppressed after SLC7A5 gene knockdown *in vitro* (**Figure 7C**). Furthermore, we conducted Matrigel-based assays and scratch experiments to evaluate the invasion and migration capabilities, respectively, of cells after SLC7A5 knockdown. We found that exogenous downregulation of SLC7A5 significantly decreased the invasive and migratory abilities of both MDA-MB-231 and 4T1 cells (**Figures 7D–H**). In summary, the inhibition of SLC7A5 expression reduced the proliferation, migration, and invasion ability of TNBC cells.

**Figure 8:**
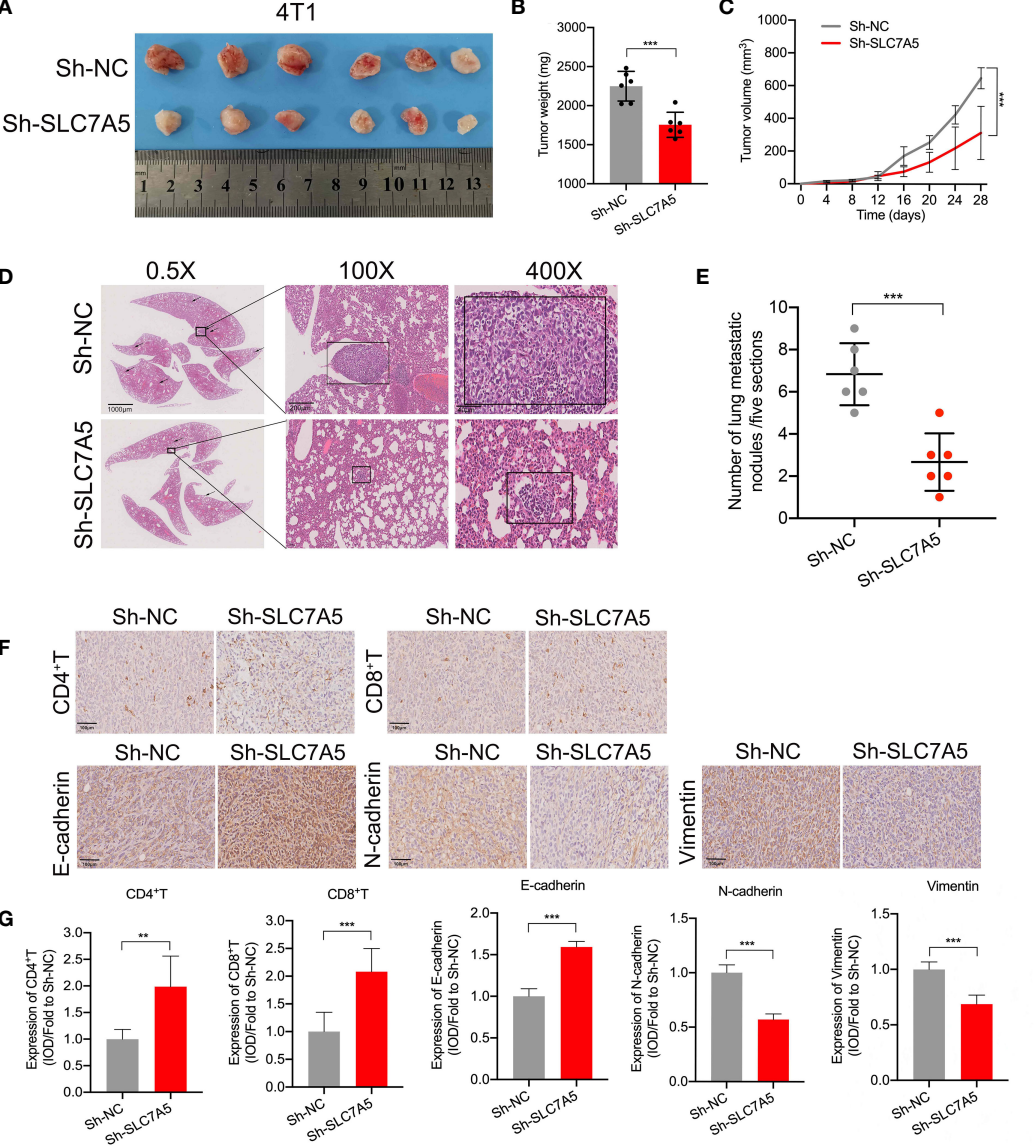
SLC7A5 promotes TNBC cell metastasis and inhibits immune cell infiltration. **(A)** Images showing subcutaneous tumors of the fat pat in BALB/c mice in the sh-NC (n=6) and sh-SLC7A5 (n=6) groups. **(B, C)** Weight and volume of tumors removed from sacrificed mice in the sh-NC and sh-SLC7A5 groups. **(D, E)** Representative images and statistical analysis of in HE-stained metastatic nodules in lung sections (scale bars: 1000 µm, 200 µm and 40 µm). **(F, G)** IHC staining and levels of EMT markers, including E-cadherin, N-cadherin and Vimentin, and the degree of immune cell infiltration, including CD4^+^ T and CD8^+^ T cells (scale bar: 100 µm). TNBC, triple-negative breast cancer; HE, hematoxylin and eosin; IHC, immunohistochemistry; EMT, Epithelial-mesenchymal transition. The asterisks represent the statistical significance as determined by the *P* value ( **P< 0.01,***P< 0.001).

In addition, to better understand how SLC7A5 knockdown hampered TNBC progression, we explored the regulatory effect of SLC7A5 on the expression of key genes associated with the EMT *in vitro*. SLC7A5 downregulation decreased N-cadherin, vimentin, and snail expression and increased E-cadherin expression, indirectly illustrating the tumor-promoting effect of SLC7A5 on the EMT (**Figures 7I-J**, **Figures S4A. B**). Using an *in vivo* lung cancer metastasis model, we established orthotropic breast tumor-bearing BALB/c mice by inoculating 4T1 cells into the fat pad to investigate the role of SLC7A5 in cancer metastasis (**Figure 8A**). SLC7A5 downregulation led to a relatively lower tumor weight and volume and a decreased number of metastatic nodules formed in pulmonary tumor lesions compared with those in the negative control mice (**Figures 8B–E**). Subsequent IHC staining confirmed that the expression levels of N-cadherin and Vimentin in lung tumor lesions were significantly decreased in the SLC7A5-downregulated group compared with the control group (**Figures 8F, G**). Moreover, we compared immune cell infiltration and found that the downregulation of SLC7A5 induced the abundance ofCD4^+^ T and CD8^+^ T cells. Collectively, these results suggested that SLC7A5 knockdown attenuates tumor progression and immune suppression in TNBC.

### Blockade of PD-1 and SLC7A5 activity synergistically increased immune cell infiltration and inhibited tumor progression in TNBC

**Figure 9:**
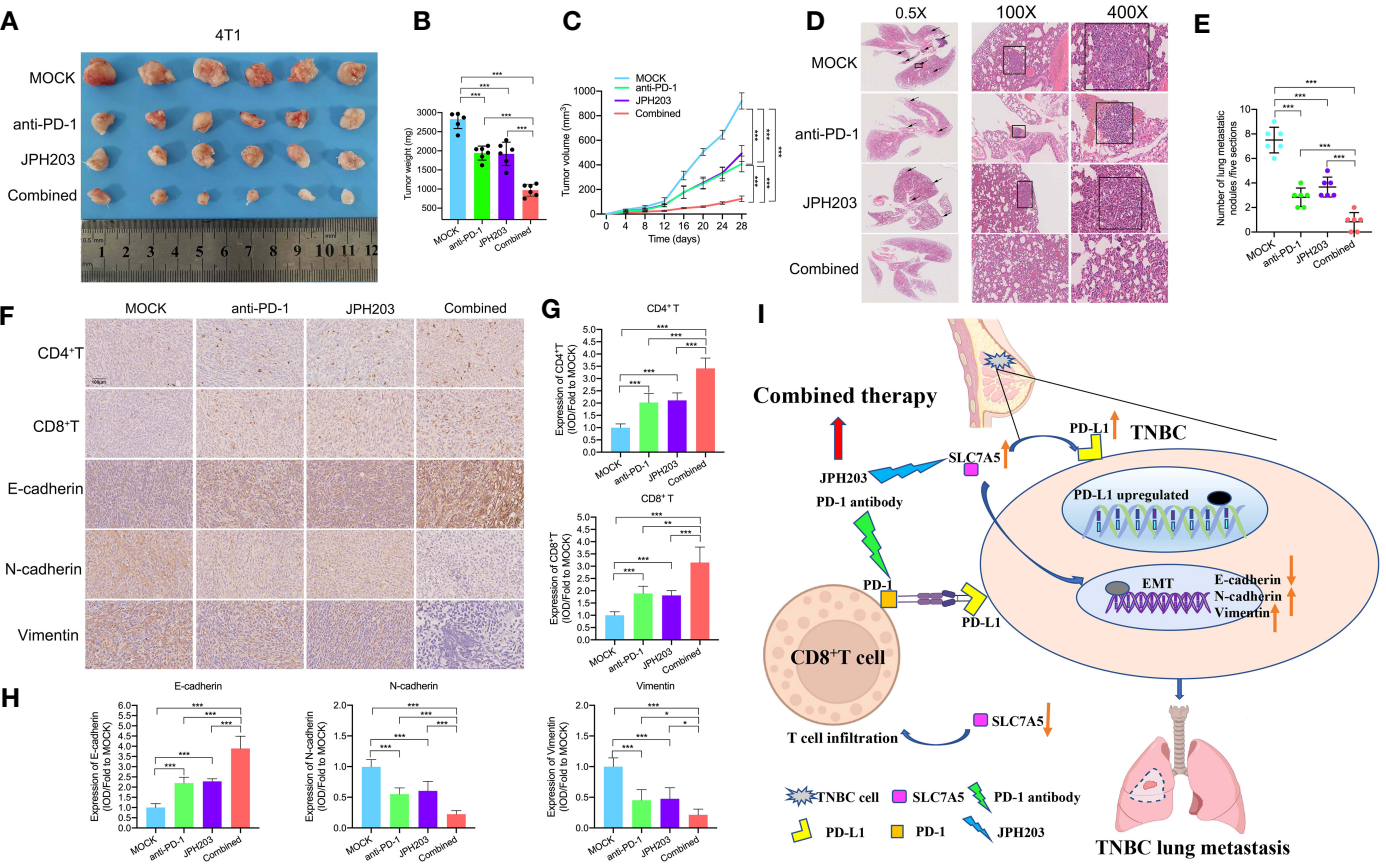
Targeting PD-1 and SLC7A5 synergistically increases immune cell infiltration and enhances antitumor efficacy in TNBC. **(A)** Images showing subcutaneous tumors of the fat pat in BALB/c mice in the MOCK (n=6), anti-PD-1 (n=6), JPH203 (SLC7A5 inhibitor) (n=6) and combined (n=6) groups. **(B, C)** Weight and volume of tumors removed from BALB/c mice in the MOCK, anti-PD-1, JPH203 and combined groups. **(D, E)** Representative images and statistical analysis of metastatic nodules in HE-stained lung sections in these four groups (scale bar: 1000 µm, 200 µm and 40 µm). **(F, H)** IHC staining and the levels of EMT markers, including E-cadherin, N-cadherin and Vimentin, and the degree of immune cell infiltration, including CD4^+^ T and CD8^+^ T cells, in the four groups (scale bar: 100 µm). **(I)** Schematic representation showing the mechanism by which SLC7A5 was more expressed more highly in TNBC cells; notably high SLC7A5 promoted cell metastasis, and the combined use of an anti-PD-1 antibody and the SLC7A5 inhibitor JPH203 was used to effectively treat TNBC. PD-1, programmed cell death protein 1; TNBC, triple-negative breast cancer; HE, hematoxylin and eosin; IHC, immunohistochemistry; EMT, epithelial-mesenchymal transition. The asterisks represent the statistical significance as determined by the *P* value (*P< 0.05, **P< 0.01,***P< 0.001).

To investigate the therapeutic efficacy and immune microenvironment remodeling realized through targeted SLC7A5 and anti-PD-1 agent combination therapy *in vivo*, we established 4T1 subcutaneous fat pat tumor models. Tumor-bearing mice were randomly assigned to four groups and administered vehicle, an anti-PD-1 antibody, JPH203 or the combination of both every two days for 28 days. The results showed an enhanced antitumor effect in both the JPH203 group and the anti-PD-1 group, and in the combination group, the antitumor effect was greater than that in either monotherapy group or the control group (**Figure 9A**). Consistent with the changes in tumor volumes, the mean tumor weights had substantially decreased in the combined group compared with those in the monotherapy groups or the control group (**Figures 9B, C**). Additionally, lung metastasis nodules in each group were counted. The number of lung metastasis nodules was decreased after targeted treatment with SLC7A5 and/or PD-1. The combination therapy significantly decreased the number of lung metastasis nodules compared with the effect of either monotherapy (**Figures 9D, E**). We also investigated whether targeting SLC7A5 or PD-1 inhibits the EMT or stimulates immune cell infiltration. Notably, in contrast with the monotherapy groups, the combination group showed a greater capacity to inhibit the EMT as well as induce greater CD4^+^ T-cell and CD8^+^ T-cell infiltration (**Figures 9F–H**). Therefore, we concluded that the glutamine metabolism-associated gene SLC7A5 promotes TNBC tumor growth and metastasis. The downregulation of SLC7A5 stimulates T-cell infiltration. Moreover, the present research reveals a novel therapeutic strategy in which CAA-derived CXCL8 is used to sensitize TNBC to immunotherapy. The blockade of SLC7A5 and PD-1 activity synergistically inhibited TNBC growth and lung metastasis, stimulated T cell activity, and enhanced the therapeutic effect of anti-PD-1 immunotherapy (**Figure 9I**).

## Discussion

TNBC is highly heterogeneous disease with characteristic alterations in the tumor immune microenvironment. The classification of TNBC cannot be used to meet the requirements for personalized treatment. Immunotherapy still shows considerable limitations, although its efficacy was initially promising. Metabolic reprogramming is a hallmark of tumorigenesis. Glutamine dependence is evident in tumor cells, and it facilitates the rapid growth and reproduction of tumor cells. In addition, altered glutamine metabolism contributes to a hypoxic, acidic, nutrient-depleted TME, which is detrimental to an antitumor immune response. Targeting the glutamine metabolic pathway in combination with immunotherapy is expected to be a novel therapeutic modality that can improve the prognosis of TNBC patients.

In this study, we investigated the expression of 17 GMGs between tumor tissues and normal tissues, and all these GMGs were differentially expressed. Considering the GMG identified, we established a glutamine metabolism typing model and assigned TNBC patients to two subtypes, Cluster A and Cluster B. Compared with those in Cluster A, the patients in Cluster B showed more abundant immune cell infiltration, higher immune checkpoint expression, higher TME scores, and better prognosis. In addition, we constructed a glutamine metabolism scoring method, GMS, to assess glutamine metabolism in TNBC patients based on five GMGs associated with TNBC prognosis. The low-GMS population was associated with more-aggressive clinicopathological features and a worsened prognosis. Moreover, the TNBC patients in the high-SLC7A5 group were associated with more-aggressive clinicopathological features and worsened prognosis, and the downregulation of SLC7A5 attenuated TNBC progression and immunosuppression. Moreover, *in vivo* experiments confirmed that the blockade of SLC7A5 activity combined with immunotherapy synergistically increased immune cell infiltration and enhanced targeted therapeutic efficacy.

Glutamine is an important nonessential amino acid (22). In tumor cells, “glutamine addiction” is evident (23). Glutamine enters cells through specific transport proteins and is converted into glutamic acid via the action of glutaminase, and it is further transformed into α-KG and enters the tricarboxylic acid cycle (TCA), providing a necessary nitrogen source for the biosynthesis pathways in tumor cells and participating in the occurrence and development of tumors (24). Glutamine depletion is particularly pronounced in TNBC compared to other types of breast cancer, which exhibit glutamine dependence. A metabolomic analysis showed lower glutamine levels and higher glutamate levels in TNBC samples, indicating enhanced glutamine catabolism (7). In addition, amino acid transporter proteins such as SLC1A5 and SLC7A5 are overexpressed in the triple-negative breast cancer samples, enabling tumor cells to meet the high demand for amino acids (25, 26). *In vitro* experiments demonstrated that in cocultures with GS-expressing cells in glutamine-free medium, glutamine-dependent basal-like breast cancer cells showed increased viability (27). *In vivo* studies confirmed that glutaminase expression was increased in primary TNBC samples compared to other breast cancer subtypes and normal tumor tissue and showed sensitivity to glutaminase inhibitors (28). Thus, targeting the glutamine metabolism pathway is expected to provide new and personalized treatments for TNBC patients. Glutaminase inhibitors combined with mTOR inhibitors exert a synergistic effect that increases TNBC treatment efficacy (29). GLUD1 inhibitors have been shown to attenuate the proliferation of breast cancer cells in mouse models (30).

Altered glutamine metabolism affects the tumor microenvironment. Tumor cells and immune cells compete for glutamine, which leaves immune cells with an inadequate source of glutamine, affecting their antitumor immune activity. In TNBC, tumor cells compete for glutamine in the TME, leading to an inhibited antitumor immune response by tumor-infiltrating T lymphocytes, and in a GLS-deficient mouse model, limiting glutamine access to tumor cells restored tumor-infiltrating T lymphocyte activity (8). Upregulation of PD-L1 expression has been observed in kidney cancer cell lines and bladder cancer cell lines cultured in glutamine-depleted culture medium (31). Limiting glutamine uptake by tumor cells led to a decrease in GSH levels, and the restricted synthesis of GSH led to the activation of the NF-κB signaling pathway, promoting the upregulation of PD-L1 expression and inactivating cocultured T cells (14). *In vitro* studies have confirmed that inhibition of glutamine utilization by tumor cells combined with anti-PD-L1 antibodies synergistically induced T-cell-mediated cancer cell death (14). In a mouse tumor model, treatment targeting glutamine metabolism administered in combination with an anti-PD-L1 monoclonal antibody increased the antitumor immune response. Blockade of PD-L1 has been used to treat TNBC, but the efficacy of immunotherapy is still limited, and various strategies are being investigated to increase treatment response rates. The distribution of activated tumor-infiltrating lymphocytes among T cells is a key limiting factor to the response to anti-PD-L1 therapy in patients with TNBC. According to the glutamine metabolism clustering established in our study, immune checkpoints were upregulated and an immune cell infiltration was more abundant in Cluster B, suggesting that glutamine metabolism genes exert a certain effect on the TME. Combining glutamine inhibitors with an anti-PD-L1 antibody promotes glutamine uptake by immune cells and increases the immune response. Various glutamine inhibitors have been shown to block glutamine uptake by tumor cells and enhance the killing effect of T cells (15). A potent glutamine transporter protein inhibitor, V9032, targets ASCT2, inhibits glutamine uptake and promotes the infiltration of tumor-infiltrating lymphocytes into the tumor core (8, 32).

SLC7A5, as an important amino acid transporter, is essential for the uptake of amino acids by tumor cells. According to previous studies, SLC7A5 is expected to become an emerging therapeutic target for breast cancer. SLC7A5 is an antiporter protein that exports intracellular glutamine and promotes leucine uptake. Overexpression of SLC7A5 has been widely observed in tumor tissues. High SLC7A5 mRNA expression levels are associated with poor prognosis in several cancer types, including breast cancer (33). Amino acid transport and glycolysis are both important MYC-controlled metabolic signaling pathways, and the proliferation of human mucosal-associated invariant T cells relied on a MYC-SLC7A5-glycolysis metabolic axis (34). SLC7A5 could mediate large neutral amino acids uptake in activated T cells, but the SLC7A5-null T cells failed metabolically reprogram and could not undergo clonal expansion or effector differentiation (35). SLC7A5 expression is positively correlated with hypoxia-related gene expression in ER-positive breast cancers, such as HIF1A and MKI67 (36). In addition, SLC7A5 is associated with endocrine resistance in luminal-type breast cancer cells. There are fewer studies on the role of SLC7A5 in the development of TNBC, drug resistance or prognosis. Our study suggests that SLC7A5 promotes the proliferation and metastasis of TNBC cells by promoting the tumor-related EMT. Similar to our results, the findings of Kurozumi et al. demonstrated that high SLC7A5 expression led to the identification of a subset of invasive breast cancers with high aggressiveness and a high tumor immune response (37). Inhibition of SLC7A5 inhibits protein synthesis by downregulating the mTORC1 signaling pathway (38) and mobilizing the general amino acid control pathway in cancer cells. The mainstream inhibitor currently used against SLC7A5 is JPH203, which is a compound that was designed based on the conformational relationships among SLC7A5 ligands. It is an inhibitor with one of the highest affinities for SLC7A5 and exerts a less profound effect on other transporter proteins and has induced no significant toxicity in preclinical studies. JPH203 has been shown to induce the inhibition of tumors established with human tumor cell lines and in mouse models (39, 40). Our study suggests that targeting SLC7A5 shows the potential to be a new therapeutic modality in TNBC and that combining it with anti-PD-L1 antibody therapy may synergistically increase the efficacy of both treatments.

Although our study confirmed a role for amino acid metabolism in TNBC, some limitations affected our results. Our findings are based on a comprehensive bioinformatics analysis and preliminary experimental verification, but more detailed experiments are still needed to investigate specific mechanisms of action. In addition, the information on amino acid metabolism based on patients in the real world and clinical information obtained from enough samples needs to be combined. Clinical trials conducted in the future may help promote the clinical application of targeted metabolism therapy.

## Conclusions

In summary, we explored the function of glutamine metabolism genes in TNBC and identified two different subtypes. Given the importance of metabolic reprogramming in tumors, targeting the glutamine metabolism pathway is expected to be a novel therapeutic modality for TNBC. Glutamine metabolism affects the TME, and immunotherapy combined with targeted glutamine metabolism inhibitors shows a synergistic effect that enhances therapeutic efficacy. SLC7A5, as an important amino acid transporter that shows the potential to become a prognostic marker for TNBC and to be a novel therapeutic target. Our research contributes to a better understanding of the relationship between glutamine metabolism and TNBC, providing guidance for the clinical management of TNBC patients.

## Data availability statement

The datasets presented in this study can be found in online repositories. The names of the repository/repositories and accession number(s) can be found in the article/**Supplementary Material**.

## Ethics statement

The studies involving humans were approved by the Medical Ethics Committee of Ruijin Hospital, Shanghai Jiao Tong University. The studies were conducted in accordance with the local legislation and institutional requirements. The participants provided their written informed consent to participate in this study. The animal studies were approved by the Medical Ethics Committee of Ruijin Hospital, Shanghai Jiao Tong University. The studies were conducted in accordance with the local legislation and institutional requirements. Written informed consent was obtained from the owners for the participation of their animals in this study. Written informed consent was obtained from the individual(s) for the publication of any potentially identifiable images or data included in this article.

## Author contributions

KS, ZW, XC, and RH were the core members who conceived and designed the whole article. RH, HW, JH, JW, OH, JRH, WC, and YL collected the clinical samples together for tissue microarray and clinical samples. RH, JH, and ZW analyzed the raw data. RH and HW wrote manuscript draft in the beginning. ZW, KS, and XC revised the final version of the manuscript. All authors have contributed to the study and approved the final version of the manuscript before the process of submitting.

## Supplementary material

Supplementary Figure 1 Kaplan−Meier curve showing 14 glutamine metabolism genes in the merged databases (TCGA-BRCA and GSE42568). **(A−K)** The 8 glutamine metabolism genes that were negatively correlated with survival. **(L-Q)** The 6 glutamine metabolism genes that were positively correlated with survival.

Supplementary Figure 2 Univariate Cox regression analysis and survival analysis based on the GMS. **(A)** HR and p value from the univariate Cox regression analysis of glutamine metabolism genes (criterion: p value< 0.05). **(B)** Survival analysis of patients with breast cancer in the high-GMS and low-GMS groups. Red indicates low expression, and blue indicates high expression. GMS: Glutamine metabolism score.

Supplementary Figure 3 Cell communications between SCL7A5-positive, -negative cancer cells and remained cell types in TNBC. **(A)** Cell-cell interaction plots of communication intensity between SCL7A5-negative (left), -positive (right) and other cell types. **(B)** Potential signal pathways between SCL7A5-positive/SCL7A5-negative cancer cells and other cells.

Supplementary Figure 4 Statistical analysis of Western blot assays **(A)** and immunofluorescence assay **(B)** of EMT marker expression after SLC7A5 knockdown in the MDA-MB-231 and 4T1 cell lines.
