# Supplementary figures and images for: Targeting glutamine metabolic reprogramming of SLC7A5 enhances the efficacy of anti-PD-1 in triple-negative breast cancer
Source: Front Immunol. 2023 Sep 4;14:1251643. doi: 10.3389/fimmu.2023.1251643 (PMC10507177; doi:10.3389/fimmu.2023.1251643)

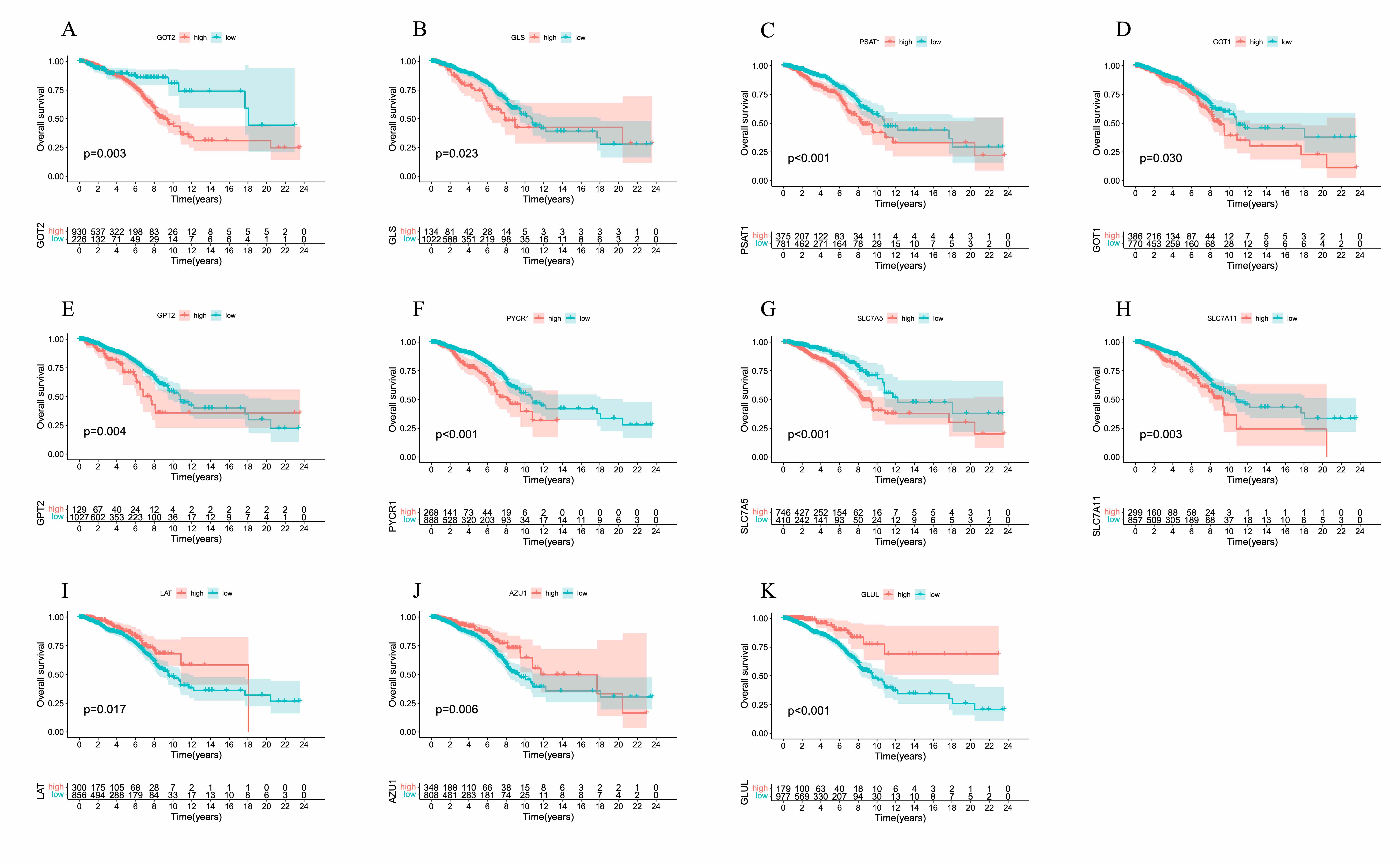

Supplement: Supplementary Figure 1 — Kaplan−Meier curve showing 14 glutamine metabolism genes in the merged databases (TCGA-BRCA and GSE42568). (A−K) The 8 glutamine metabolism genes that were negatively correlated with survival. (L-Q) The 6 glutamine metabolism genes that were positively correlated with survival. [file Image_1.jpg]

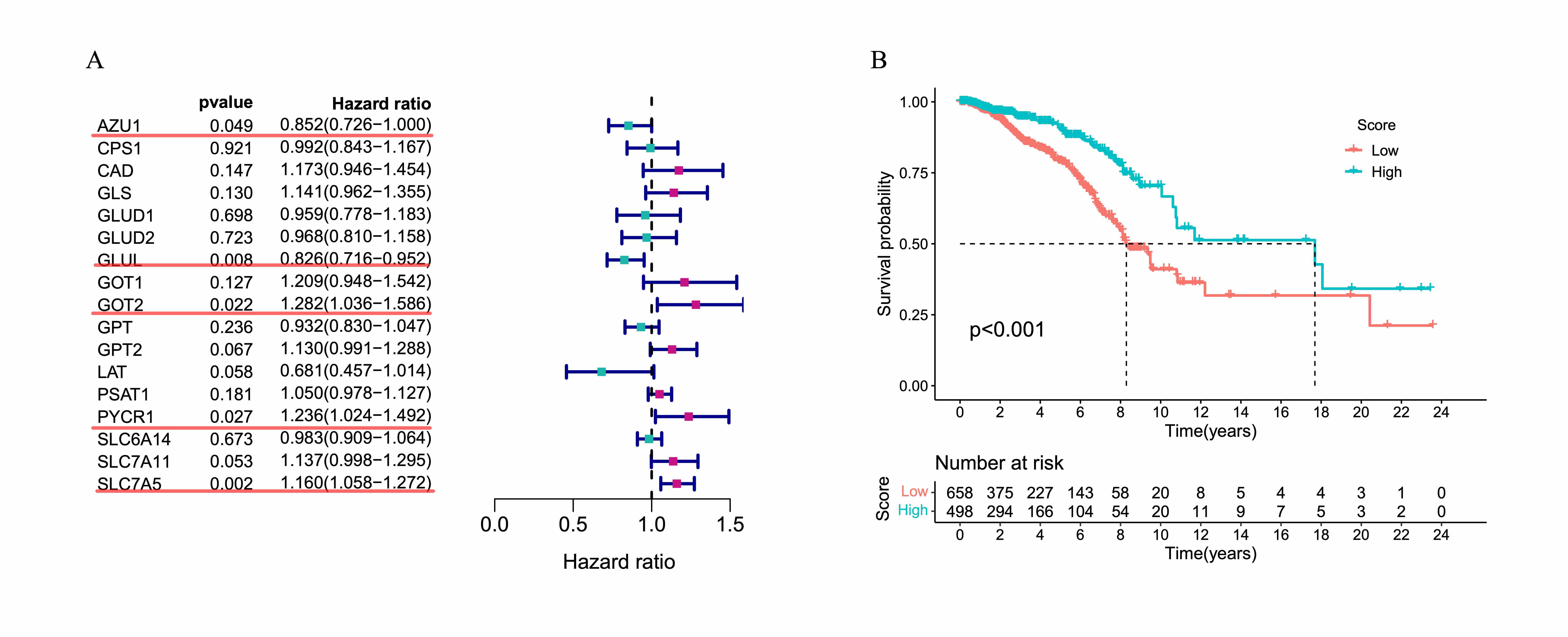

Supplement: Supplementary Figure 2 — Univariate Cox regression analysis and survival analysis based on the GMS. (A) HR and p value from the univariate Cox regression analysis of glutamine metabolism genes (criterion: p value< 0.05). (B) Survival analysis of patients with breast cancer in the high-GMS and low-GMS groups. Red indicates low expression, and blue indicates high expression. GMS: Glutamine metabolism score. [file Image_2.jpg]

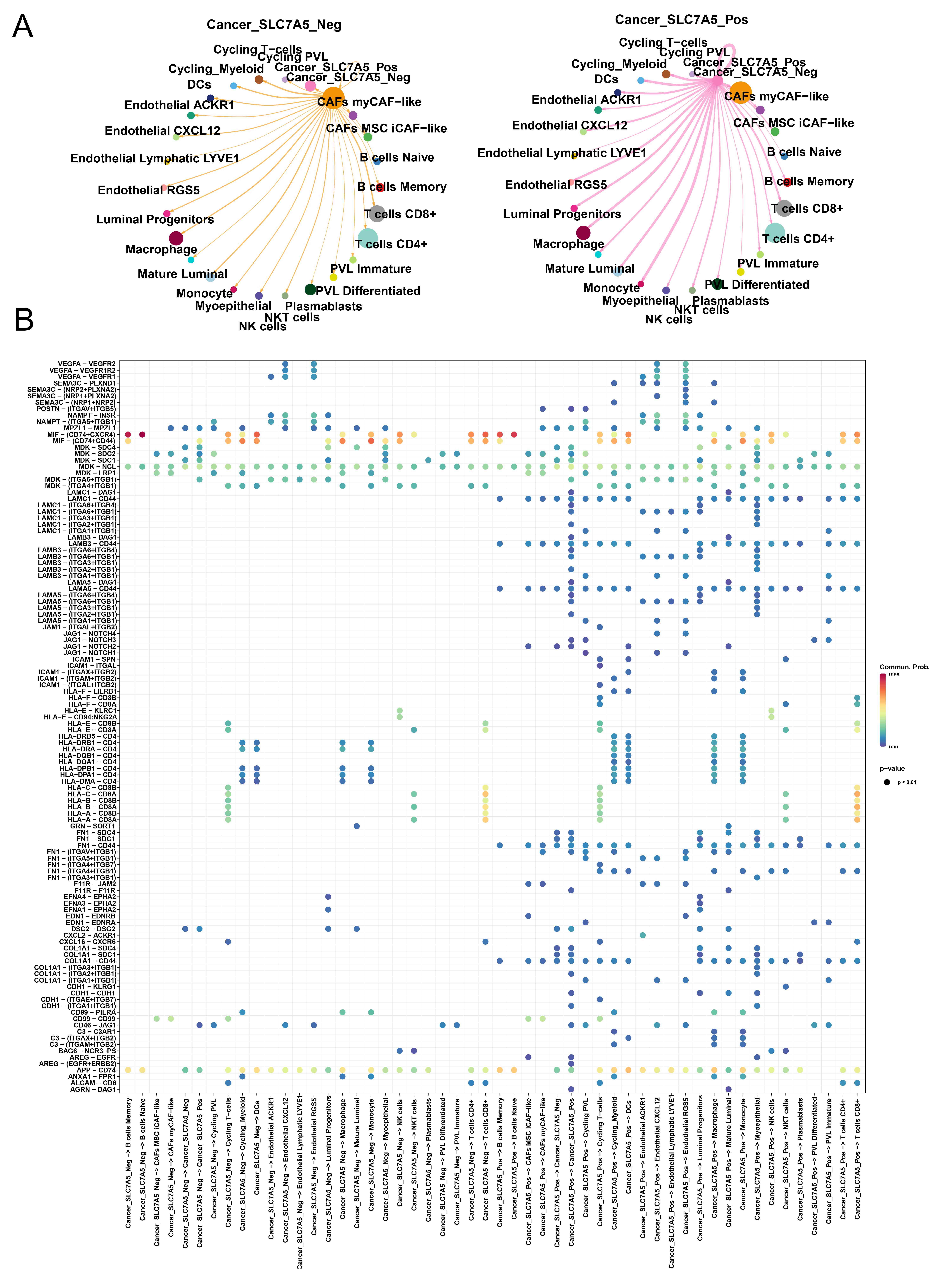

Supplement: Supplementary Figure 3 — Cell communications between SCL7A5-positive, -negative cancer cells and remained cell types in TNBC. (A) Cell-cell interaction plots of communication intensity between SCL7A5-negative (left), -positive (right) and other cell types. (B) Potential signal pathways between SCL7A5-positive/SCL7A5-negative cancer cells and other cells. [file Image_3.jpg]

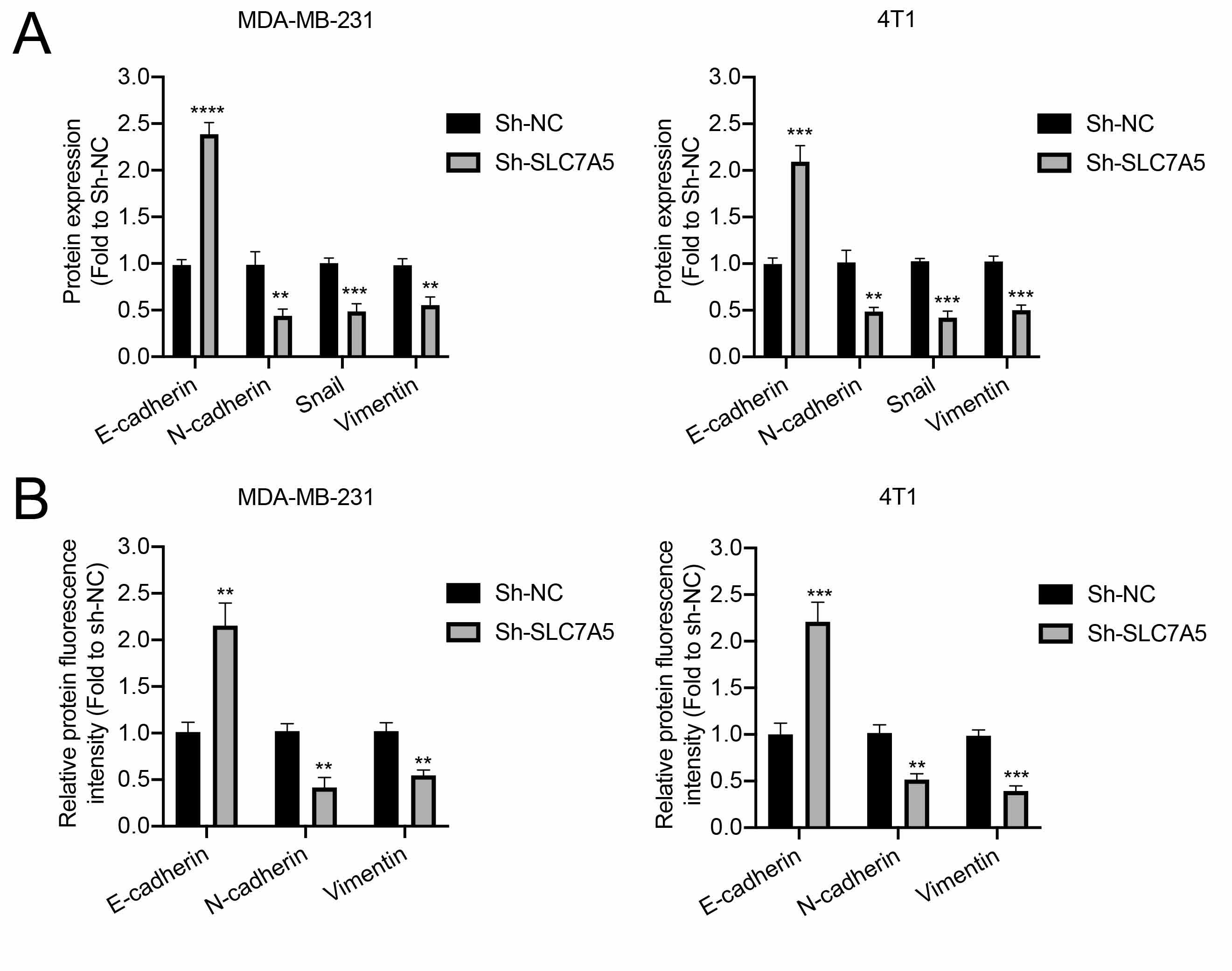

Supplement: Supplementary Figure 4 — Statistical analysis of Western blot assays (A) and immunofluorescence assay (B) of EMT marker expression after SLC7A5 knockdown in the MDA-MB-231 and 4T1 cell lines. [file Image_4.jpg]
